# Precarity, agency and trust: Vaccination decision-making in the context of the UK asylum system

**DOI:** 10.1016/j.ssmqr.2024.100515

**Published:** 2025-06

**Authors:** Anna Deal, Maha Salloum, Sally E. Hayward, Alison F. Crawshaw, Felicity Knights, Jessica Carter, Isra Al-Sharabi, Reem Yahia, Stephanie Fisher, Beatriz Morais, Oumnia Bouaddi, Lucy Jones, Anna Miller, Sandra Mounier-Jack, Sally Hargreaves

**Affiliations:** a The Migrant Health Research Group, City St George's, University of London, UK; b Faculty of Public Health and Policy, London School of Hygiene and Tropical Medicine, London, UK; c The Population Health Research Institute, City St George's University of London, UK; d The Vaccine Institute, City St George's University of London, UK; e Mohammed VI University of Sciences and Health, Casablanca, Morocco; f Doctors of the World UK, London, UK

## Abstract

**Background:**

Individuals living in initial asylum accommodation are at increased risk of vaccine-preventable disease, yet confidence in vaccination may be low in these settings. Our aim was to understand the influence of experiences within the UK asylum system on vaccine confidence and decision-making from a sociological perspective.

**Methods:**

In-depth semi-structured interviews were carried out on views and experiences around vaccination (09/2020-08/2021) with individuals seeking asylum or having recently been granted asylum (<10 years in the UK). Interviews were audio-recorded, transcribed and analysed in NVivo 12 using a reflexive thematic analysis through an inductive approach.

**Results:**

25 participants were interviewed (mean age: 37 years, mean time in UK: 6 years, 72% female), of whom 13 were living in asylum accommodation at the time of interview. Analysis generated three main themes: 1) the detrimental impact of trauma and fear, both within the UK asylum system and prior, on perceptions of risk and vaccination decisions, 2) the effect of marginalisation, discrimination and neglect within the asylum system on an individual's trust and 3) the structural violence and restricted agency imposed on asylum seekers and its effect on ability and motivation to vaccinate. Past trauma or negative experiences since arriving in the UK, such as feeling forced to receive ‘invasive’ healthcare interventions in asylum accommodation may lead to distrust, increased perception of danger and avoidance of perceived ‘risks’ such as vaccination. Participants described how their struggle to cover basic necessities, social isolation and precarious living conditions imposed by the asylum system left them with more pressing priorities than vaccination. Participants who perceived that they had been cared for with empathy in the healthcare system or who described feeling empowered to make their own decision about vaccination often suggested they would be likely to accept vaccination if offered.

**Conclusions:**

Asylum seekers and refugees have often experienced substantial trauma and precarity and have a lack of agency directly imposed on them by the asylum system. These factors are likely to impact trust and decision-making around vaccination, with some also representing systemic or structural barriers to accessing services. Formative experiences in the UK are key to establishing trust in healthcare settings; a trauma-informed approach should be central in developing vaccination interventions for these groups, particularly in asylum accommodation.

## 1 Introduction

The scale of forced migration globally has increased dramatically in the last decade. The UNHCR estimated that at the end of 2022, there were 108.4 million forcibly displaced people worldwide as a result of conflict, violence, persecution or human rights violations, including 35.3 million refugees and 5.4 million asylum seekers (UNHCR). People seeking humanitarian protection have often experienced trauma associated with the circumstances that have forced them to flee their country of origin and often make dangerous migration journeys (Stevens & Ciftci, 2022), leaving them at increased risk of infectious diseases, mental health challenges and restricted access to healthcare, such as vaccination. A systematic review exploring outbreaks of vaccine-preventable diseases in Europe reported a high number of outbreaks among migrants in temporary refugee camps or accommodation centres, linked to missed vaccines in their home country and due to their mobility, lack of access to vaccination systems, and poor living conditions before and after arrival (Deal et al., 2021a). To compound this risk, previous research suggests that some migrant groups with precarious immigration status, including asylum seekers, have low confidence in vaccination and low uptake, with hesitancy often strongly rooted in cultural, historical and religious context among different groups (Deal et al., 2021b).

**Fig. 1:**
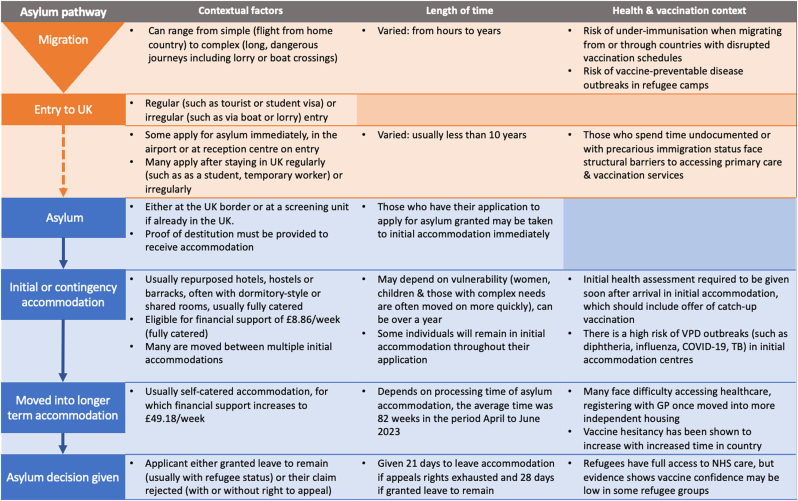
An overview of the UK asylum pathway and the social, health and vaccination contexts relevant to each stage.

In the UK, the total asylum caseload has increased more than four times 2014, with the number of individuals waiting for a decision on their asylum application reaching 224,742 in June 2024 (Home, 2024). This situation has been driven by a backlog in processing applications as well as a significant increase in applications placed (Sturge, 2023). Consequently, there has been a shift towards accommodating newly arrived asylum seekers in large-scale and ‘contingency’ accommodation centres, which include repurposed hotels, hostels, or military barracks (Doctors of the World UK, 2022; The Home Office, 2023). Health challenges related to the asylum system, particularly for those resident in large-scale and contingency accommodation, have been widely reported, such as infectious and vaccine-preventable diseases, poor nutrition, lack of mental health support and difficulties accessing healthcare (Deal et al., 2023; Jones et al., 2022) (see Fig. 1). The risk of vaccine-preventable diseases has been particularly highlighted, with residents often living in cramped conditions and low immunisation coverage known to be a risk in forced migrant populations, particularly among adults, who may have missed doses as children in their home countries (Deal A, Crawshaw, & et al, 2021; Knipscheer et al., 2015). UKHSA has reported several vaccine-preventable disease outbreaks in asylum accommodation in the last two years, including diphtheria, varicella, COVID-19 and influenza (UK Health and and Security Agency, 2023), with some of these outbreaks, such as diphtheria, raising considerable operational challenges and requiring specific, costly public health responses (O'Boyle et al., 2023). These incidents highlight the risk these individuals face and the need for accessible immunisation services, particularly for adults outside of routine childhood vaccination services.

NHS Integrated Care Boards are responsible for commissioning healthcare services for asylum seekers in their area, including those in initial and contingency accommodation. This includes an initial health assessment for all residents (Jones et al., 2022), with guidelines emphasising the importance of infectious disease screening and vaccination (Knights et al., 2022; NHS, 2021). No UK-wide specifications exist to regulate the timing, content or location of these health assessments, with wide variations dependent on practice by local authority, ICB or housing provider (The Red Cross, 2024), although most are carried out soon after arrival in Home Office accommodation. While there are no minimum requirements for vaccinating newly arrived adult asylum seekers in the UK, initial health assessment guidelines recommend following the UKHSA's guidance on the vaccination of individuals with uncertain or incomplete immunisation status (Knights et al., 2022; NHS, 2021; UKHSA, 2013). For adults, this is focused on providing three doses of Td/IPV, two of MMR and one of MenACWY.

It is clear that the asylum system places potentially under-immunised individuals into a high-risk situation for infectious and vaccination-preventable diseases. However, it has been previously suggested that confidence in and uptake of vaccinations may be low among asylum seekers in the UK and other high-income countries (Deal et al., 2021b; Nichol et al., 2022; Stevens et al., 2021). Structural factors, such as living conditions associated with the asylum system, lack of transportation, low income, and limited knowledge of the local healthcare system, are likely to hinder access to vaccination in this group, particularly adults outside of routine childhood vaccination serviceS (Deal et al., 2023). Additionally, cultural, religious and contextual factors have been widely recognised as influencing vaccine confidence in migrant groups more widely (Deal et al., 2023; Gehlbach et al., 2021; Gorman et al., 2019; Gorman et al., 2020; Jenness et al., 2021; Lockyer et al., 2021; Tankwanchi et al., 2021). Trust has also been acknowledged as a key factor in vaccine confidence; previous research clearly shows that trust in healthcare systems, vaccination itself, the wider system in which an individual lives, as well as a sense of agency over healthcare decisions are all strongly tied to vaccine confidence (World Health Organization, 2022). The COVID-19 pandemic and subsequent COVID-19 vaccine roll-outs highlighted the negative effects of marginalisation, structural racism and precarity all of which are likely to be experienced by those seeking asylum (Jones et al., 2022), on trust and subsequently vaccination confidence and uptake (Crawshaw et al., 2022; Deal et al., 2023; Deal et al., 2021b; Lockyer et al., 2021; World Health Organization, 2022). While some migrant groups may have existing hesitancy around vaccination from earlier life experiences or due to vaccine hesitancy in their home country, previous research has highlighted the potential for distrust of vaccination increasing or originating after arrival in a host country, driven by social exclusion (Tankwanchi et al., 2021), structural racism or precarity in resettlement (Campeau, 2019). For example, a US study showed that among Karen refugees from Myanmar, perception of vaccine safety decreased with more time spent in the US (Truman et al., 2020). Experience of trauma or post-traumatic stress disorder (PTSD), both prevalent among asylum seekers (Knipscheer et al., 2015; Pfeiffer et al., 2022; Wylie et al., 2018), may impact on an individuals’ capacity for trust and have previously been observed to have a subsequent effect on vaccine confidence (Christou-Ergos et al., 2022a, 2022b; Milan & Dáu, 2021). For example, in an Australian study from 2022, Christou-Ergos et al. (Christou-Ergos, Leask, & Wiley, 2022) described how medical trauma may shape vaccine refusal. This study culminated in the generation of a model to show how individuals views on vaccinations may be re-evaluated as a result of coping strategies to deal with negative feelings and trauma around healthcare.

In the context of increasing numbers of forced migrants arriving in high income countries globally, it is crucial to understand the impact of asylum systems on the health of individuals seeking protection, as well as wider public health. While people seeking asylum are likely to be under-immunised, at risk of vaccine-preventable diseases (Deal A, Crawshaw, & et al, 2021; Deal et al., 2023; Deal et al., 2021a; Deal et al., 2021b), and may have low confidence in vaccination, there is little research to date on the impact of asylum systems on decision-making around vaccination and vaccine confidence. Therefore, this study aims to fill the gap in knowledge on the interplay between the conditions imposed by the UK asylum system and trust, precarity, agency and vaccination decisions.

## 2 Methods

We used a reflexive qualitative approach to explore the effect of the UK asylum system on individuals' perspectives and decision-making around adult vaccinations (outside of the routine childhood vaccination schedule), and to explore opportunities for improving trust and confidence in vaccination among those in the asylum system. We adopted a mainly inductive approach throughout the research study, to suit the exploratory and novel nature of the research questions and the evolving context of the COVID-19 pandemic and ongoing changes to national asylum policy. Ethics was granted by St George's, University of London Research Ethics Committee (REC, 2020.0058).

### 2.1 Participant recruitment

We sought to interview individuals currently seeking asylum in the UK, although our inclusion criteria also included those recently granted refugee status (<10 years in the UK) through the asylum route. Participants were recruited using purposive and snowball sampling, aiming to recruiting participants from a broad range of nationalities, age groups and cultural backgrounds. Adverts for the study and participant information sheets were circulated to 20 UK-based migrant support groups and on social media groups (Facebook and WhatsApp) set up for asylum seekers in the UK. Those who showed interest in taking part were contacted by email or telephone, depending on their preference. We offered a preliminary phone call to either read the information sheet verbally or explain in short form, dependent on participant preference, and to give participants the opportunity to ask questions about the study. Interpreters were available for both the preliminary phone call and the interview itself. If consent was given, participants were offered an interview time at their convenience and asked their preference of medium (phone call or online voice/video call through Microsoft Teams or Zoom). Due to COVID-19 pandemic restrictions at the time, in-person interviews were not offered. Participants were compensated with an online shopping voucher as per INVOLVE NIHR criteria for participant involvement in research studies (£37 for an up to 90-min interview) (NIHR, 2016).

### 2.2 Semi-structured interview design and data collection

Topic guides were developed through iterative cycles by the research team comprising AD, SH, SMJ, AC, SEH (academic researchers) and JC, FK (General Practitioners), using a narrative chronological approach, which involves structuring questions in a way that encourages participants to construct narrative and stories of their lived experiences (Nasheeda et al., 2019). Topic guides were informed by research addressing challenges in designing qualitative research with culturally diverse and marginalised groups (Merry et al., 2011). During piloting, we engaged with migrant representatives from the Migrant Health Research Groups’ Project Board to explore suitability and acceptability of the topic guide. Topic guides were centred around questions asking broadly about their experiences of healthcare and vaccination in the UK as adults, individual journeys to the UK and experiences in the asylum system. In addition, we sought to understand individual vaccination decision-making processes and factors influencing acceptance or rejection of all or specific vaccines.

39 semi-structured interviews were carried out between September 4, 2020 and August 31, 2021 by AD, MS and SEH. In-depth semi-structured interviews lasted around 30–90 min. The researchers, who identify as female and White British (AD, SEH) or Syrian (MS) all come from an academic background. Researchers made notes during or directly after each interview, and the lead researcher (AD) kept a reflexive journal throughout the data collection and analysis phases, which included input from field notes provided by MS and SEH. Interviews were conducted in English, Bengali (n = 1) and Tamil (n = 1). Interviews were audio-recorded, and the English language content transcribed verbatim (using Way with Words transcription service); transcripts were checked for accuracy and anonymised.

### 2.3 Data analysis

We used an inductive approach to data analysis, given the lack of previous research exploring the impact of experiences within the asylum system on vaccination decision-making. Analysis followed the reflexive thematic analysis approach described and recently updated by Braun and Clarke (Braun and Clarke, 2006, 2019; Braun et al., 2022). After a phase of data immersion, during which transcripts and field notes were intensively read, a phase of initial, open coding was done on ten transcripts by the primary researcher (AD), combining both latent and semantic coding. This was used to develop ideas for preliminary themes, which were brought to key members of the research team for discussion and reflection. Based on this period of reflection, a coding framework, consisting of mostly latent codes, was developed around the preliminary themes and used to code all remaining transcripts in NVivo 12. A second period of reflection by the primary researcher (AD), with input from other team members, led to the refinement of preliminary themes into final themes and sub-themes. In the themes arising, we recognised a level of congruence with an existing model developed by Christou-Ergos et al. (Christou-Ergos, Leask, & Wiley, 2022), which describes how experiences of medical trauma shape vaccine refusal. We therefore used this model as a framework to structure our themes, developing a novel model to describe how experiences in the UK asylum system impact on vaccination confidence and decision-making.

## 3 Results

### 3.1 Participant demographics

The majority of participants were currently in the asylum system (76%) and the remaining participants had been granted refugee status through the asylum route in the last ten years. Around half (52%) of participants reported living in Home Office-provided accommodation at the time of interview. Participants were mostly female (72%) and were of a wide range of ages, with a median age of 37 years (range 22–59). On average, our participants had lived in the UK for 5 years and came from a wide range of countries of origin (see Table 1). Participants reported a range of different routes into the asylum system, with some having claimed asylum on arrival at an airport or ports, and others having arrived in the UK on other visas, sometimes overstaying, and later claimed asylum, although this data was not formally collected.

**Table 1:** Demographics of study participants.

Characteristic	n (%)
**Migrant status**	
Asylum seeker	19 (76%)
Refugee	6 (24%)
**Living in Asylum accommodation**	
Yes	13 (52%)
No longer	6 (24%)
Information not shared	6 (24%)
**Time since arrival in the UK (years), mean**	**5.1**
0–2	7 (28%)
3–5	5 (20%)
6–10	12 (48%)
Unknown, <10	1 (4%)
**Age in years, mean (range)**	**37.2 (22–59)**
18–30	3 (12%)
30–35	8 (32%)
36–40	7 (28%)
Over 40	7 (28%)
**Gender**	
Female	18 (72%)
Male	7 (28%)
**WHO Region of origin**	
African Region (Mauritius, Nigeria, Uganda, Zimbabwe, other/unknown)	7 (28%)
Eastern Mediterranean Region (Afghanistan, Egypt, Iraq, Pakistan, Palestine, Syria)	8 (32%)
European Region (Albania, Kyrgyzstan, Turkey, Ukraine)	4 (16%)
Region of the Americas (Venezuela)	1 (4%)
South-East Asian & Western Pacific Region (India, Sri Lanka)	5 (20%)

### 3.2 Thematic analysis and model

Asylum seekers often arrive in the UK with a history of traumatic experiences. They subsequently face intersecting stressors in the UK, such as precarity, lack of agency and marginalisation, many as a direct result of the asylum system and policies surrounding it. This can have a major impact on ability to trust, mental health and risk perception, affecting individuals’ perceptions and decision-making around healthcare and vaccination. We generated three themes to describe how living within the UK asylum system affects how individuals may perceive vaccination and make vaccination decisions for themselves and their families; 1) the impact of trauma and fear, both before and within the UK asylum system, on perceptions of risk and vaccination decisions, 2) the effect of marginalisation, discrimination and neglect in the asylum system on trust and 3) the structural violence and restricted agency imposed on asylum seekers and its effect on ability and motivation to vaccinate.

**Fig. 2:**
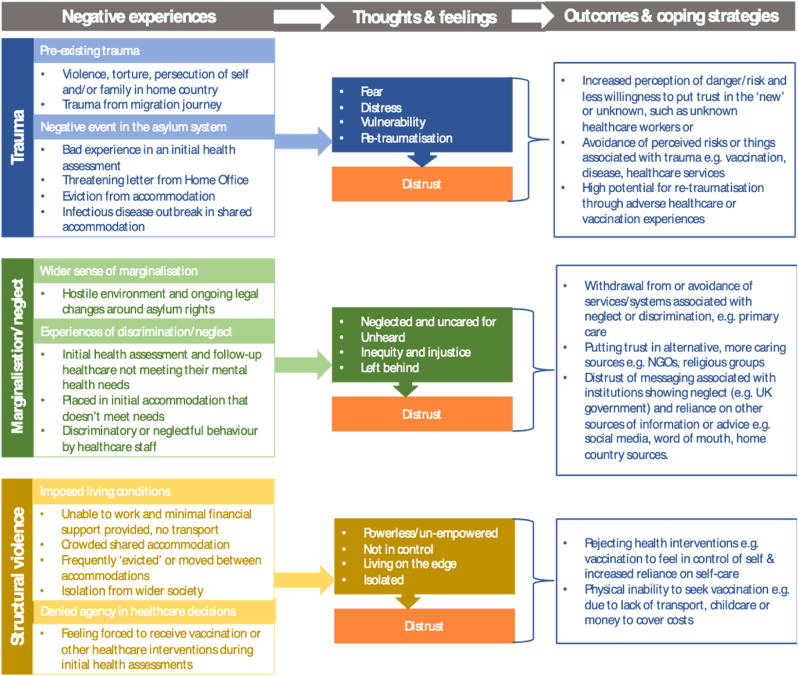
*The impact of trauma, structural violence, marginalisation and neglect on vaccination decisions and outcomes, based on* Christou-Ergos et al. (Christou-Ergos, Leask, & Wiley, 2022).

Based on these themes and a model previously developed by Christou-Ergos et al. (Christou-Ergos, Leask, & Wiley, 2022), which describes the impact of medical trauma on vaccine refusal, we developed a model (see Fig. 2). This model illustrates our three themes and how the key factors in each of them (trauma, marginalisation and neglect, structural violence) lead to thoughts and feelings that culminate in distrust. It also describes the coping mechanisms and outcomes relating to vaccination that may occur as a result.

#### 3.2.1 The effect of living through trauma and fear on perceptions of risk and vaccination decisions

Many participants recounted experiencing traumatic events prior to their arrival in the UK, such as war, persecution, and torture. The majority of participants discussed ongoing suffering from mental health conditions, such as PTSD, depression, and anxiety, which had often been exacerbated during the COVID-19 pandemic. It was generally implied by participants that many asylum seekers arrive in the UK in a state of fear, which heightened their perception of risk and wariness of the unknown, making vaccination more likely to be perceived as a threat.

“I was tortured back in my country and I fled my country and I came here and was very worried if the police find out about me or wherever I am. So I had to isolate myself […] I don’t want to be like a rapid test experience […] I don’t want to be the first one who would be experience it [COVID-19 vaccine] and get all the negative side from it […] I can’t go deeper but that's what makes me really fear and scared”

P4

“They’re just fleeing from the country where there’s war, there’s harassment, there’s political instability, there’s poverty […] because of these situations that they’ve faced […] they cannot concentrate, because of the stress, they lose confidence in themselves […] Your mind is just thinking about maybe, you’ve lost your mum, you’ve lost your family, lost your children, left them in that country, trying to flee to a safe place. Then, they start telling you about this vaccine, you cannot concentrate, you don’t understand.”

P12

After arrival in the UK and entering the asylum process, many participants discussed the continuation, and in some cases, the exacerbation of their state of trauma and fear, often as a direct result of the asylum system and the lack of clarity around their future. Many linked this to a lowered trust in services and authorities, including healthcare:

‘When I reached the UK, I thought, oh, I reached, everything will be fine […] But then I started having different problems. If I faced in my country physical and psychological abuse, violence, in this country I’m facing psychological abuse’

P13

“When it comes to helping or getting information of asylum seekers […] they have been through a lot. They come in the country that they don’t trust much and they won’t tell you much”

P16

“I know some of my, maybe three, friends that have passed away because they are afraid to go to the hospital”

P19

Some participants spoke about specific challenging or traumatic events they had experienced since seeking asylum in the UK, including negative experiences of healthcare and vaccination. One participant described the fear they felt whilst residing in initial accommodation while feeling forced to accept an unknown vaccination during an initial health assessment:

“I went through severe traumatic experiences with all the things, with the war that started back home and I was frightened. And then we came to register with the GP and they said you need to do the vaccination today. I didn't know what vaccination, why, why all of a sudden? […] They didn't even ask me for that [vaccination history] and then all of a sudden they decided to do it. And it was frightening.”

P7

Another participant described how a negative experience with a blood test affected their trust in primary care, and emphasised how coming from background of trauma made them quicker to re-evaluate their trust in a person or system as a result of negative experiences:

“Why I would prefer to get it [vaccination] somewhere else [than primary care], is because the time I went for blood test, the woman that took my blood … Some of them don’t care […] She was difficult to get my vein […] She just shook anywhere that blood come out, and later I have reaction on that hand […] Because I came from a place of abuse and neglect, I’m always mindful to anyone that will give me ill treatment”

P22

When asked about the barriers they faced to vaccination, some participants directly identified fear as a barrier to vaccination, particularly COVID-19 vaccination, relating this to their situation of being alone in an ‘unknown’ country. Several mentioned a fear of dying in the UK and not knowing what would happen, how their family in their home country would be informed, often mentioning their children who would be left alone in an unstable situation in the asylum system with no-one to care for them. This fear was brought up by some as reason to avoid vaccination, driven by the fear of serious side effects from vaccines, but others viewed it as a reason to get vaccinated to prevent serious illness.

“I’m alone, I’m only single parent, and I look after my baby. If something happened with me, who will come to my baby […] I have an appointment that day [to get a vaccine], and I said, I already cancelled this one. Because I’m scared, if me put injection, who look after my baby?”

P25

“I’m the only adult in the house, so it made me to be in a very difficult situation. I just feel that I’m going to die, or something is going to happen to me [as a result of vaccination], and my children are just going to be stranded.”

P10

Although many described lowered trust in vaccination, authorities or healthcare as a result of past trauma and uncertainty around their asylum applications, some participants rather discussed fear in relation to disease, with several particularly bringing up worries around the risks of living in shared accommodation. These participants often expressed very positive views around vaccination, considering it as a step towards safety.

“So if any danger [i.e disease], if I feel a danger and then I can immune myself I’m happy to. So, all these years I’m moving because of the safety, because of life.”

P1

“They will not like to contract any disease while they are here [in shared, initial accommodation]”

P15

### 3.3 Trauma, fear and the importance of messaging

Misinformation or anecdotes that play on fear, particularly traumatic stories of people in similar situations, appeared to have a powerful influence on some participants and their trust in healthcare and healthcare professionals.

“I was reading another article, a lady who trusted the GP so much, but the GP was just a scam, he was stealing people’s moneys […] He was making all the old people die and then he would take the money for that person”

P12

This was linked by one participant to an existing fear and lack of trust on arrival in the UK, which exacerbates the effect of traumatic anecdotes:

“I see when people just arrive, and I was the same, they lack trust of anyone who represents the country […] People have heard a lot of stories […] I myself have heard the story about my friend, who moved to Switzerland, and she was pregnant and the baby died inside and they didn't do anything. So she said they did it intentionally, so my friend will not have babies. And that thing sticks in my head”

P7

Participants also discussed the fear that information that appears to come from an ‘official’ or government-related source might provoke, often due to traumatic experiences of receiving letters written in a threatening tone from the Home Office. This may lead to an association of any official communication with fear, including letters, texts or reminders from the NHS around vaccination. This was described by the participant below:

‘You have fear about anything, about any letters, about any SMS messages, because it’s a very uncertain situation. It’s a precarious situation, you don’t know what’s going to happen next. Any letters coming from Home Office, or any SMS, is creating a fear first”

P8

#### 3.3.1 ‘They don't care about us': the effect of marginalisation, discrimination and neglect in the asylum system on trust

This theme describes how marginalisation, discrimination or a feeling of being neglected and uncared about can affect both an individual or an entire community's trust, widely recognised as a key factor in vaccination decisions (Larson, 2020). Many of our participants associated trust with feeling that a person, authority or system has their best interests at heart, and feeling respected and understood by that entity. In reverse, those perceived as neglectful, uncaring or opposing their interests were likely to not be trusted.

“Of course, I would be listening to the doctor or the nurse that is talking to me about healthcare. But the Home Office agents that hamper me and then brought me into that army barracks, I would tend to think, I’m a little bit reluctant to [hear] what you’re saying”

P16

The majority of our participants felt that the ‘system’ (UK institutions and wider society) does not care about them, listen to them or treat them equally to UK citizens, leading, in some cases, to low trust in healthcare providers and vaccination services. This appeared to be particularly true for participants who had recently arrived in the UK and were in the early stages of the asylum process. Negative experiences within the asylum system, in particular, were brought up to describe a general sense of marginalisation, discrimination and neglect in the country. Similarly, experiences of neglect or discrimination in the healthcare system were associated with lowered trust in future healthcare interventions, such as vaccination, offered to them.

“I don’t think they’re listening enough […] People cannot just make up things […] There is something that is chasing the man from his place and make him to come seek refuge in another place. […] They were saying they had this, they had that and they [Home Office/authorities] never believe them. Now they’re dead […] The person say I’m going through this, I’m going through that. You don’t want to listen”

P22

“What I felt and many people are feeling, when you go to hospital and you are an asylum person, they feel like they don’t do enough for you. They don’t care the same, if you are an asylum seeker. We feel, and I can’t say for sure that’s happening, but we feel that”

P18

Some related this low trust in authorities to fear around vaccination, as they did not trust the intention behind vaccination campaigns:

“Once a government now confuses [them], they [will] not trust the government, he who doesn’t know English very well […] they can’t believe these are vaccinations [i.e. they might be a way to control the population etc]”

P11

In the UK, an asylum seeker's first interaction with healthcare services will often be during initial health assessments after moving to Home Office accommodation, where vaccines are usually offered. However, many of our participants reported feeling that health assessments and the care they receive on arrival doesn't follow their needs or priorities, generating a sense of neglect. Nearly all participants struggled with mental health, and in some cases other health conditions, and felt abandoned to deal with these alone.

*“Nobody asks you how are you feeling after you came into this country, are you anxious, are you depressed, are you suicidal, are you sleeping OK? […] nobody cares, [if] maybe you have a chronic disease, you have a mental issue, if you’ve been immunised, how your immune system is and how well you are nourished*”

P15

Some participants felt that authorities are only pursuing their own interests when supposedly providing services to those in the asylum system. For example, using healthcare appointments to find out why asylum seekers came to the UK, or using them as ‘guinea-pigs’ for testing vaccinations.

“I feel like nobody pays attention to my medical condition at all. Because what is interesting for them, what is important for authorities, is why I come to this country”

P13

“Why do they have to try the [COVID-19] vaccine in the most vulnerable groups? we wouldn’t be the guinea pigs, we wouldn’t like, just because we are vulnerable or because we, nobody cares for us … no, we wouldn’t do that”

P15

In contrast, positive experiences of healthcare or authorities, such as being treated well by healthcare professionals, feeling cared for, empathised with and treated equally was often linked with trust and a greater willingness to vaccinate. Positive experiences with NHS maternity services were often described as an example. It was also notable that routine vaccinations for their children and vaccinations during pregnancy were generally viewed in a more positive light, potentially due to positive associations with maternity services and routine NHS services for young children, and less association with asylum settings.

“They [NHS] listened to me and they listened to my mental health […] they don’t ask whether you are a student or an asylum seeker. They just respect you as a human […] I didn’t say no [to vaccination], because they’re just doing everything”

P3

“if a GP really shows empathy and interest for you, of course you will feel, oh this particular GP is really interested in my situation and he is worried about me and he wants to help me […] I will rely on the doctor and I will rely on the vaccine and I will know that the doctor is doing me a favour and preventing disease in the future”

P15

In general, individuals or organisations who were perceived as having the participant's best interests in mind were more likely to be trusted. This varied widely across participants and included religious groups, friends and families, charities, and in some cases, the NHS or specific healthcare professionals. Where participants felt they had received more care, support or understanding from religious groups, charities or other non-healthcare entities, or had experienced neglectful behaviour within the healthcare system, many chose to place their trust in the entities who they felt cared for them when making vaccination or healthcare decisions:

“Actually, I’m a new believer, and before, I was okay. In my country we’ve been having all vaccinations, I was vaccinated all my life. But as soon as I’m a believer and I come to this country, I started attending church [..] our church leaders, they’re all saying to us not to be vaccinated. Even vaccination against flu, they say we shouldn’t take it”

P13

A key issue brought up by nearly all participants was the isolation and loneliness they experienced during the asylum process, often as a direct result of how they are housed, and in many cases exacerbated by the pandemic. Some discussed how isolation hindered their ability to access healthcare information, leaving them in small circles of knowledge, more susceptible to misinformation:

“I have some true friends, they’re also asylum seeker. We talk about ourselves, our problems”

P11

“They have a variety of kind of news coming from different places. I think it’s from their culture and from their networks, because they don’t actually get the expert information. The way to get information is from friends […] misinformation is so easy to come in these communities”

P8

#### 3.3.2 Structural violence: the conditions imposed on asylum seekers and how they affect ability and motivation to vaccinate

This theme describes the structural violence that the asylum system imposes on individuals within it, directly restricting their agency and power and imposing physical conditions on them that may hinder their ability to seek healthcare and vaccination. We have divided this theme into two subthemes. The first explores the effect of the removal of agency and how this affects trust in and interactions with vaccination. The second subtheme examines the physical living conditions imposed on asylum seekers and how this affects their ability and motivation to accept vaccination.

### 3.4 Removal of power, liberty and agency by the asylum system and its effect on trust

Many seeking asylum arrive in the UK in a position of relatively low agency, due to often limited English language or literacy skills, economic challenges and legal position, suffering from mental health conditions, and unfamiliarity with local systems. Those who arrive in a position of less agency and power, such as those who don't speak English, are more likely to feel and be discriminated against or neglected by systems in the UK. As discussed in the previous theme, this is likely to have a major impact on their trust in these systems.

“They [friends who don’t speak English] get frustrated very easily because they don’t feel like their concerns are being heard, because when they don’t know how to speak the language, and then they go, and then they don’t understand, maybe, the person who’s on the reception is being hostile?”

P12

After arrival, the rules and living conditions imposed by the asylum system directly remove nearly all remaining liberty from individuals, as they are given specific accommodation, with no choice of where they are placed and no means of transport.

“We don’t even have the capability to even give ourselves what we really want to give ourselves, the healthcare or the care that we really want to give ourselves or our families. We just depend on the government.”

P10

In this context, where individuals are entirely dependent on the state, vaccination, particularly when administered shortly after arrival in Home Office accommodation or without adequate explanation, may seem forced. Individuals may feel unable to refuse vaccination due to their limited agency in such a position. This sense of lack of empowerment coupled with the negative emotions of discrimination and abuse surrounding healthcare was described by one participant recounting their experience of having a blood test soon after arrival:

“One lady tried to get blood from body about 20 times. Do you know what it is to try getting blood from you 20 times? It’s horrible of pain […] At that time [when recently arrived], I wasn’t empowered to say, that’s my body, you can’t do that […] I’m sure that what the lady did to me, she would never have done to English people because they know how it works. I didn’t know and I couldn’t say anything.”

P18

Another participant recounted an experience of feeling forced to receive MMR vaccinations, seemingly due to a lack of written documentation of their previous vaccination, despite having verbally informed the healthcare professional, that they had already received these vaccinations:

“I got a vaccine at child age, for measles, and here also, England, last year, they gave me the same one. […] she put it on the computer in my language and I said, I got it, this one [measles vaccine]. And then she said, no, now you have to take again […] I was worried, why they are giving me this injection?”

P21

Such experiences, particularly where the reasons for a vaccination being administered or re-administered have not been well explained (such as, in this case, UKHSA guidelines recommending re-vaccination in the case of missing documentation), can exacerbate a profound sense of lack of agency, making individuals feel powerless. We noticed a pattern that some participants appeared to reject future vaccinations, such as flu or COVID-19 vaccinations, after experiencing a sense of powerlessness around healthcare, often during initial health assessments. This rejection may represent an attempt to regain agency over their bodies and decisions. This is described by the participant below:

“[In initial accommodation] they said you need to do the vaccination today. I didn't know what vaccination, why, why all of a sudden? […] They didn't even ask me for that [vaccination history] and then all of a sudden they decided to do it. And it was frightening […] when they sent me these flu jabs, I refused all of them.”

P7

In contrast, participants who felt they had a choice over whether to get vaccinated and who received a clear explanation about it, felt a greater sense of agency and were much more likely to accept:

“I just got the flu-jab, that’s the one that I had. Yes, because the doctor was really nice, and when I got there, said, do you want the vaccine?”

P12

“They also let us choose whether to take it or not. But we prefer to take it, so we said, you know better than us”

P2

Living on the edge: ‘When you have a lot of problems, sometimes you don't care about vaccines'

This sub-theme describes the living conditions imposed on those in the asylum system and how this affects ability and motivation to get vaccinated. Many participants implied that they have ‘bigger things to worry about’ than getting a vaccination, such as financial worries, untreated mental health concerns, living in shared accommodation, being frequently relocated, and the ongoing stress of uncertainty around their asylum applications.

“When you have a lot of problems, sometimes you don’t care about vaccines or something like that, because you have other priorities”

P19

Some conditions in the asylum system pose direct barriers, such as lack of financial means or transport to get to the GP. Some participants also emphasised that not all asylum seekers have their passport with them, but many GP surgeries will require this for registration. This can create a fear around approaching primary care. This is described by the participant below:

“Some of them that say they’re afraid. And some of the GPs when you want to register, they will ask for your passport. And they don't have the passport, so they don't have the document to show”

P19

Participants talked about being frequently relocated between different accommodation centres, particularly whilst they were in the initial accommodation phase, often negatively affecting the continuity of their healthcare. This included issues receiving vaccination in cases where multiple doses are required over a specific timeframe.

“I was in a hostel, and at that time, they were almost ready to obtain help [healthcare for a specific condition], but in the meantime, we moved house, and then nothing happened”

P6

## 4 Discussion

While the asylum system puts potentially under-immunised individuals in a situation of high risk of vaccination-preventable diseases, this study reveals that it also creates an environment where many known drivers of low confidence in vaccination, such as distrust, marginalisation, isolation and restricted agency, may prosper. We observed that experiences of trauma, marginalisation and structural violence within the asylum system, particularly considering most asylum seekers are fleeing trauma, violence or persecution in their home countries, can directly influence trust, including in specific systems such as healthcare. Negative experiences of healthcare and vaccination in initial health assessments after arrival in Home Office accommodation risk re-traumatising individuals arriving in the UK in a position of relatively low agency and may result in coping strategies that involve longer-term rejection of or distrust in vaccination. Given the rising numbers of asylum seekers arriving in high-income countries, many coming from regions with disrupted vaccination systems, a holistic, trauma-informed approach to vaccination within asylum systems is key. This will be crucial for fostering long-term trust, ensuring the inclusion of these groups in vaccination initiatives, and promoting vaccine equity.

Currently, processes for initial health assessments and vaccination for asylum seekers varies widely by local authority, ICB and housing provider (The Red Cross, 2024), making such recommendations more challenging to implement. There is a need for health bodies in the UK to provide an official recommendation for healthcare providers and regulatory bodies, such as ICBs, to ensure consistency in provision. We recommend that these guidelines should be based on a trauma-informed approach. Table 2 expands on recommendations specific to the design and delivery of vaccination campaigns for asylum seekers, based on the findings of this research.

**Table 2:** Recommendations for applying a trauma-informed approach to the design and delivery of vaccination services for asylum seekers residing in initial and dispersed accommodation centres, based on the six pillars of trauma-informed practice listed by the UK Office for Health Improvement & Disparities (OHID) (Office for Health Improvement and Disparities, 2022a).

Pillar of trauma-informed practice	Recommendations for design and delivery of vaccination services
**Safety**	• Healthcare professionals (HCPs) should take steps to ensure the individual feels safe and has given informed consent before administering a vaccination, to avoid association of fear and trauma with vaccines; • Aim to prevent re-traumatisation by providing an option to delay vaccination for those suffering from trauma and unable to give clear and informed consent; • Ensure invite letters or communication around vaccination for those seeking asylum do not resemble ‘official’ government messages in style or language to avoid generating fear; • Information campaigns and doctor-patient communication should put an emphasis on vaccination as a route to safety and give clear information about the dangers of VPDs and how vaccination protects against these; • Consideration for providing separate appointments for vaccinations and blood tests, where this could be beneficial

**Trustworthiness**	• As a minimum, HCPs should give a clear explanation, with qualified interpreters, of the vaccination(s) they are offering, the rationale (including why they may offer a vaccine without knowing the patient's vaccination history) and what the process will involve; • Information campaigns around vaccination for asylum seekers could focus messaging on showing care, such as: ‘You are being offered this vaccination because we care that you don't get sick’; • Vaccine programmes should be embedded within services that are able to address the range of health and wellbeing needs a person has, including mental health; • Initial health assessments (IHAs) should be developed with a holistic approach, to not appear too focused on vaccines and to ensure asylum seekers are set up with other support needed as a priority.

**Empowerment & Choice**	• Information about the vaccination(s) offered should be provided at multiple points and in different formats (e.g. multilingual information leaflets, social media, workshops or educational meetings in accommodation centres); • HCPs should emphasise that vaccination is a choice and should give adequate time to address any concerns; • Qualified interpreters should be available during all appointments involving vaccination, to make sure information and choices around vaccination are fully understood.

**Collaboration & Cultural Consideration**	• The format, content and delivery of IHAs and related information campaigns should be co-designed with people with lived experience of the asylum system and relevant voluntary, religious or community groups, to ensure they are responsive to the needs of these groups; • Information and communication around vaccination(s) should be culturally tailored for different communities and available in a wide range of languages; • Access to gender- and culture-responsive services should be ensured, for example, a choice of female or male vaccinator should be offered to meet religious or cultural requirements

A significant body of research indicates that trust in healthcare providers, vaccination and political systems is strongly correlated with vaccine acceptance (Tankwanchi et al., 2021). We have shown that experiences of trauma, both before and after arriving in the UK to seek asylum, can influence an individual's ability to trust, potentially affecting long-term vaccine confidence. While little research prior to this study has examined how trauma affects vaccine confidence among asylum seekers and refugees specifically, an Australian study has previously observed individuals who have experienced medical-related trauma subsequently rejecting vaccination as a coping mechanism (Christou-Ergos, Leask, & Wiley, 2022). A US study has also shown that mothers with a history of PTSD had significantly less confidence in COVID-19 and childhood vaccines (Milan & Dáu, 2021), and several studies have linked experiencing significant trauma or PTSD with developing mistrust of institutions generally, including healthcare services and providers (Gobin & Freyd, 2014; Klest et al., 2019; Milan & Dáu, 2021). The prevalence of PTSD among asylum seekers and refugees has been estimated to be as high as 31% (Blackmore et al., 2020) and a large majority of asylum seekers have experienced multiple traumatic or adverse life events (Knipscheer et al., 2015; Pfeiffer et al., 2022), suggesting that, considering the findings of our study, this may be a significant factor influencing vaccine confidence in this group. A sense of restricted agency has also previously been shown to affect vaccine confidence; our study found that some individuals, feeling unable to refuse or question vaccinations during initial health assessments, may reject future vaccinations as a way to regain a sense of control. Marginalisation, isolation and a feeling of neglect are further factors that existing evidence shows can impact trust in vaccination, particularly among migrant and ethnic minority groups (Deal et al., 2023; Deal et al., 2021b). This issue is particularly evident in our study in terms of how well an individual's needs (particularly mental health) were met in the initial stages of the asylum system on their trust in healthcare professionals, the NHS and vaccination. A multitude of reports indicate that, whilst variable by region (Nezafat Maldonado et al., 2022; Pathway, 2023), the service asylum seekers receive in the UK often neglects their needs, vulnerabilities and health (Guma et al., 2021; Jones et al., 2022; Nellums et al., 2018).

Trauma-informed practice is a relatively recent concept, grounded in an understanding of the complex impact that trauma has on an individual's world view, trust and relationships. It is designed to support the development of services that promote safety, trust and empowerment, and prevent re-traumatisation (Bloom & Farragher, 2013; Homes & Grandison, 2021). The UK Office for Health Improvement and Disparities has listed the six key principles of trauma-informed practice, based on the original definition from the United States Substance Abuse and Mental Health Services Administration (SAMHSA) as safety, trust, choice, collaboration, empowerment and cultural consideration (Office for Health Improvement and Disparities, 2022b). Trauma-informed practice is beginning to be recognised as an important tool for designing and delivering healthcare services, particularly around mental health, for groups likely to suffer high prevalence of PTSD and trauma, such as refugees and asylum seekers (Im & Swan, 2020; Kenny et al., 2022; Miller et al., 2019; Wylie et al., 2018). However, using a trauma-informed approach to design vaccination services and interventions to increase vaccine confidence has to date not been explored in practice, despite emerging research suggesting this concept (Christou-Ergos, Wiley, et al., 2022; Gordon et al., 2023; Jee et al., 2023). In the context of the UK asylum system, using trauma-informed practice for mental health provision in initial health assessments is recommended, but recommendations do not consider how this approach could be expanded to other areas of the initial health assessment, such as vaccination (Knights et al., 2022). Our findings suggest that using a trauma-informed approach to both the delivery and design of vaccination services for asylum seekers, both in and beyond initial health assessments, may be key to developing long term trust and vaccine confidence in these groups.

While this study is the first to investigate the effect of the asylum system on vaccination decisions and has provided valuable insight, several limitations must be recognised. Our analysis was done with a reflexive approach, and therefore it is important to consider how the position and demographics may influence both participant responses and how we interpret the narratives they shared. The research team was mostly non-migrant and all non-asylum seeking, meaning they are likely to have been seen as ‘outsiders’ by the participants' and would have approached the analysis and narrative generation from an outsider perspective. While this may have limitations, such as less familiarity and trust, several benefits have also been previously observed such as more perceived freedom to discuss topics that may be associated with stigma in some communities, such as mental health. It is also important to recognise that most of our participants spoke a conversational level of English, and didn't ask for an interpreter when offered, suggesting they are a group of asylum seekers who may be in a position of more agency compared with many who do not speak English, whose views are less represented in this research.

In conclusion, we have found that the trauma and precarity asylum seekers and refugees have often experienced, as well as the lack of agency directly imposed on them by the asylum system, are likely to impact trust and decision-making around vaccination. Formative experiences in the UK, such as initial health assessments in asylum accommodation, are key to establishing trust; a trauma-informed approach could be central in designing and delivering vaccination interventions and messaging for these groups, particularly in asylum accommodation.

## CRediT authorship contribution statement

**Anna Deal:** Writing – review & editing, Writing – original draft, Project administration, Methodology, Investigation, Formal analysis, Data curation, Conceptualization. **Maha Salloum:** Writing – review & editing, Investigation. **Sally E. Hayward:** Writing – review & editing, Methodology, Investigation. **Alison F. Crawshaw:** Writing – review & editing, Methodology, Formal analysis. **Felicity Knights:** Writing – review & editing, Formal analysis. **Jessica Carter:** Writing – review & editing, Formal analysis. **Isra Al-Sharabi:** Writing – review & editing, Formal analysis. **Reem Yahia:** Writing – review & editing, Formal analysis. **Stephanie Fisher:** Writing – review & editing, Formal analysis. **Beatriz Morais:** Writing – review & editing, Formal analysis. **Oumnia Bouaddi:** Writing – review & editing, Formal analysis. **Lucy Jones:** Writing – review & editing, Formal analysis. **Anna Miller:** Writing – review & editing, Formal analysis. **Sandra Mounier-Jack:** Writing – review & editing, Supervision, Conceptualization. **Sally Hargreaves:** Writing – review & editing, Supervision, Funding acquisition, Conceptualization.

## Declaration of competing interest

The authors declare that they have no known competing financial interests or personal relationships that could have appeared to influence the work reported in this paper.

## References

[bib1] Blackmore R. (2020). The prevalence of mental illness in refugees and asylum seekers: A systematic review and meta-analysis. PLoS Medicine.

[bib2] Bloom S.L., Farragher B. (2013).

[bib3] Braun V., Clarke V. (2006). Using thematic analysis in psychology. Qualitative Research in Psychology.

[bib4] Braun V., Clarke V. (2019). Reflecting on reflexive thematic analysis. Qualitative Research in Sport, Exercise and Health.

[bib5] Braun V., Clarke V., Hayfield N. (2022). ‘A starting point for your journey, not a map’: Nikki Hayfield in conversation with Virginia Braun and Victoria Clarke about thematic analysis. Qualitative Research in Psychology.

[bib6] Campeau K.L. (2019). Vaccine barriers, vaccine refusals: Situated vaccine decision-making in the wake of the 2017 Minnesota measles outbreak. Rhetoric of Health & Medicine.

[bib7] Christou-Ergos M., Leask J., Wiley K.E. (2022). How the experience of medical trauma shapes Australian non-vaccinating parents' vaccine refusal for their children: A qualitative exploration. SSM - Qualitative Research in Health.

[bib8] Christou-Ergos M., Wiley K.E., Leask J., Shapiro G.K. (2022). Traumatic events and vaccination decisions: A systematic review. Vaccines.

[bib11] Deal A H.S., Crawshaw A.F. (2021). The immunisation status of UK-bound refugees, january 2018 – october 2019: A retrospective, population-based cross-sectional study. The Lancet Public Health.

[bib62] Crawshaw A.F., Farah Y., Deal A., Rustage K., Hayward S.E., Carter J., Majeed A. (2022). Defining the determinants of vaccine uptake and undervaccination in migrant populations in Europe to improve routine and COVID-19 vaccine uptake: a systematic review. The Lancet Infectious Diseases.

[bib13] Deal A. (2021). Strategies and action points to ensure equitable uptake of COVID-19 vaccinations: A national qualitative interview study to explore the views of undocumented migrants, asylum seekers, and refugees. Journal of Migration and Health.

[bib12] Deal A., Crawshaw A.F., Carter J., Knights F., Iwami M., Darwish M., Hargreaves S. (2023). Defining drivers of under-immunization and vaccine hesitancy in refugee and migrant populations. J Travel Med..

[bib15] Deal A., Halliday R., Crawshaw A.F., Hayward S.E., Burnard A., Rustage K., Carter J., Mehrotra A., Knights F., Campos-Matos I., Majeed A., Friedland J.S., Edelstein M., Mounier-Jack S., Hargreaves S. (2021). Migration and outbreaks of vaccine-preventable diseases in Europe. The Lancet Infectious Diseases.

[bib16] Deal A., Hargreaves S., Lau K., Zmroczek-Sterenberg M., Robinson S. (2023).

[bib17] Doctors of the World UK (2022).

[bib18] Gehlbach D. (2021). COVID-19 testing and vaccine hesitancy in latinx farm-working communities in the eastern coachella valley. Research Sq.

[bib19] Gobin R.L., Freyd J.J. (2014). The impact of betrayal trauma on the tendency to trust. Psychological Trauma: Theory, Research, Practice, and Policy.

[bib20] Gordon A.C.T., Crenstil C., Mamluk L. (2023). Attitudes and experiences of asylum seekers and refugees to the COVID-19 vaccination: A qualitative study. BJGP Open.

[bib21] Gorman D.R., Bielecki K., Willocks L.J., Pollock K.G. (2019). A qualitative study of vaccination behaviour amongst female Polish migrants in Edinburgh, Scotland. Vaccine.

[bib22] Gorman D. (2020). Comparing vaccination hesitancy in Polish migrant parents who accept or refuse nasal flu vaccination for their children. Vaccine.

[bib23] Guma T. (2021).

[bib24] Home Office. (2024). How many cases are in the UK asylum system?.

[bib25] Homes A., Grandison G. (2021).

[bib26] Im H., Swan L.E. (2020). Capacity building for refugee mental health in resettlement: Implementation and evaluation of cross-cultural trauma-informed care training. Journal of Immigrant and Minority Health.

[bib27] Jee S., Conn A.-M., Manly J.T. (2023). Let's stop the pain: A trauma-informed care approach to pediatric vaccination. Clinical Pediatrics.

[bib28] Jenness S.M., Aavitsland P., White R.A., Winje B.A. (2021). Measles vaccine coverage among children born to Somali immigrants in Norway. BMC Public Health.

[bib29] Jones L. (2022).

[bib30] Kenny M.A., Grech C., Procter N. (2022). A trauma informed response to COVID 19 and the deteriorating mental health of refugees and asylum seekers with insecure status in Australia. International Journal of Mental Health Nursing.

[bib31] Klest B., Tamaian A., Boughner E. (2019). A model exploring the relationship between betrayal trauma and health: The roles of mental health, attachment, trust in healthcare systems, and nonadherence to treatment. Psychol Trauma.

[bib32] Knights F., Munir S., Ahmed H., Hargreaves S. (2022). Initial health assessments for newly arrived migrants, refugees, and asylum seekers. Bmj.

[bib33] Knipscheer J.W., Sleijpen M., Mooren T., Ter Heide F.J., van der Aa N. (2015). Trauma exposure and refugee status as predictors of mental health outcomes in treatment-seeking refugees. BJPsych Bulletin.

[bib34] Larson H.J. (2020).

[bib35] Lockyer B. (2021). Understanding COVID-19 misinformation and vaccine hesitancy in context: Findings from a qualitative study involving citizens in Bradford, UK. Health Expectations.

[bib36] Merry L. (2011). Improving qualitative interviews with newly arrived migrant women. Qualitative Health Research.

[bib37] Milan S., Dáu A.L.B.T. (2021). The role of trauma in mothers' COVID-19 vaccine beliefs and intentions. Journal of Pediatric Psychology.

[bib38] Miller K.K., Brown C.R., Shramko M., Svetaz M.V. (2019). Applying trauma-informed practices to the care of refugee and immigrant youth: 10 clinical pearls. Children.

[bib39] Nasheeda A., Abdullah H.B., Krauss S.E., Ahmed N.B. (2019). Transforming transcripts into stories: A multimethod approach to narrative analysis. International Journal of Qualitative Methods.

[bib40] Nellums L.B., Rustage K., Hargreaves S., Friedland J.S. (2018).

[bib41] Nezafat Maldonado B., Armitage A.J., Williams B. (2022). Variation in initial health assessment of unaccompanied asylum-seeking children: A cross-sectional survey across England. BMJ Paediatr Open.

[bib42] NHS (2021).

[bib43] Nichol A.A., Parcharidi Z., Al-Delaimy W.K., Kondilis E. (2022). Rapid review of COVID-19 vaccination access and acceptance for global refugee, asylum seeker and undocumented migrant populations. International Journal of Public Health.

[bib44] NIHR (2016).

[bib45] O'Boyle S. (2023). National public health response to an outbreak of toxigenic <em>Corynebacterium diphtheriae</em> among asylum seekers in England, 2022: A descriptive epidemiological study. The Lancet Public Health.

[bib46] Office for Health Improvement and Disparities (2022).

[bib47] Office for Health Improvement and Disparities (2022).

[bib48] Pathway (2023).

[bib49] Pfeiffer E. (2022). Traumatic events, daily stressors and posttraumatic stress in unaccompanied young refugees during their flight: A longitudinal cross-country study. Child and Adolescent Psychiatry and Mental Health.

[bib50] Stevens A., Besana M., van Nispen tot Pannerden C., Sheik F. (2021). Covid-19 vaccine confidence in UK refugees, asylum seekers, and undocumented migrants. BMJ Opinion.

[bib51] Stevens A., Ciftci Y. (2022). Public health and human rights must be prioritised over inhumane immigration policies. BMJ.

[bib52] Sturge G. (2023).

[bib53] Tankwanchi A.S., Bowman B., Garrison M., Larson H., Wiysonge C.S. (2021). Vaccine hesitancy in migrant communities: A rapid review of latest evidence. Current Opinion in Immunology.

[bib54] The Home Office (2023).

[bib55] The Red Cross (2024). Delivering with dignity: A framework for strengthening commissioning and provision of healthcare services for people seeking asylum. The Red Cross.

[bib56] Truman T. (2020). Beliefs and experiences about immunization among refugees resettled in the United States from the Thailand-Myanmar (Burma) border. International Journal of Health Promotion and Education.

[bib57] UK Health and Security Agency (2023).

[bib58] UKHSA (2013).

[bib59] UNHCR. Reguee Data Finder, <https://www.unhcr.org/refugee-statistics/>(.

[bib60] World Health Organization (2022).

[bib61] Wylie L. (2018). Assessing trauma in a transcultural context: Challenges in mental health care with immigrants and refugees. Public Health Reviews.

